# Leveraging the glymphatic and meningeal lymphatic systems as therapeutic strategies in Alzheimer’s disease: an updated overview of nonpharmacological therapies

**DOI:** 10.1186/s13024-023-00618-3

**Published:** 2023-04-20

**Authors:** Douglas A. Formolo, Jiasui Yu, Kangguang Lin, Hector W. H. Tsang, Haining Ou, Georg S. Kranz, Suk-Yu Yau

**Affiliations:** 1 Department of Rehabilitation Sciences, Faculty of Health and Social Sciences, The Hong Kong Polytechnic University, 11 Yuk Choi Road, Hung Hom, Kowloon, Hong Kong, S.A.R, China; 2 Research Institute for Smart Ageing (RISA), The Hong Kong Polytechnic University, Hong Kong S.A.R, China; 3 Mental Health Research Center (MHRC), The Hong Kong Polytechnic University, Hong Kong S.A.R, China; 4 Department of Affective Disorders, The Affiliated Brain Hospital of Guangzhou Medical University, Guangzhou, Guangdong Province China; 5 School of Health and Life Sciences, University of Health and Rehabilitation Sciences, Qingdao City, Shandong Province China; 6 Department of Rehabilitation, The Fifth Affiliated Hospital of Guangzhou Medical University, Guangzhou, Guangdong Province China; 7 Department of Psychiatry and Psychotherapy, Comprehensive Center for Clinical Neurosciences and Mental Health (C3NMH), Medical University of Vienna, Vienna, Austria; 8 The State Key Laboratory of Brain and Cognitive Sciences, The University of Hong Kong, Hong Kong S.A.R, China

**Keywords:** Alzheimer’s disease, Glymphatic system, Meningeal lymphatics, Nutrition, Non-invasive brain stimulation, Traditional Chinese medicine, Physical exercise

## Abstract

Understanding and treating Alzheimer’s disease (AD) has been a remarkable challenge for both scientists and physicians. Although the amyloid-beta and tau protein hypothesis have largely explained the key pathological features of the disease, the mechanisms by which such proteins accumulate and lead to disease progression are still unknown. Such lack of understanding disrupts the development of disease-modifying interventions, leaving a therapeutic gap that remains unsolved. Nonetheless, the recent discoveries of the glymphatic pathway and the meningeal lymphatic system as key components driving central solute clearance revealed another mechanism underlying AD pathogenesis. In this regard, this narrative review integrates the glymphatic and meningeal lymphatic systems as essential components involved in AD pathogenesis. Moreover, it discusses the emerging evidence suggesting that nutritional supplementation, non-invasive brain stimulation, and traditional Chinese medicine can improve the pathophysiology of the disease by increasing glymphatic and/or meningeal lymphatic function. Given that physical exercise is a well-regarded preventive and pro-cognitive intervention for dementia, we summarize the evidence suggesting the glymphatic system as a mediating mechanism of the physical exercise therapeutic effects in AD. Targeting these central solute clearance systems holds the promise of more effective treatment strategies.

## Highlights

• The glymphatic system mediates central solute clearance.

• The meningeal lymphatics mediate central solute efflux to the cervical lymph nodes.

• Recent findings suggest both systems are involved in AD proteinopathy.

• Nonpharmacological therapies increase the activity of both systems in AD animal models.

• Increased glymphatic activity mediates physical exercise neuroprotective effects in AD.

## Background

Dementia is the leading cause of disability [1] and the largest contributing factor to dependence among the elderly [2]. With a prevalence of 5–7% for those aged over 60 and an increasingly aging population, it is expected that more than 65 million people will be living with dementia by 2030 [3]. Alzheimer’s disease (AD) is the most prevalent type of dementia, with an estimated lifetime prevalence of 3–4% [4]. It is characterized by abnormal expression of amyloid-beta (Aβ) and tau protein which, upon accumulation, aggregate and form toxic amyloid plaques and neurofibrillary tangles [5–7]. One of the biggest scientific and therapeutic challenges in AD is the elucidation of how such proteinopathy develops and how to halt its progression [8]. Although current pharmacological strategies can delay cognitive decline, disease-modifying therapeutic strategies still do not exist [5].

The challenge involving toxic protein accumulation and its halt can be attributed to an incomplete understanding of solute clearance dynamics in the central nervous system (CNS). Until recently, extracellular protein aggregates in the brain parenchyma were believed to be cleared by physiological pathways involving cellular uptake, enzymatic degradation, and transportation across the blood–brain barrier (BBB) [9, 10]. However, the recently discovered glymphatic system involving cerebrospinal fluid (CSF) bulk flow through the brain parenchyma and a comprehensive description of the meningeal lymphatic network opened a new frontier for the understanding of brain waste clearance [11–14].

The glymphatic and meningeal lymphatic systems have been implicated in the clearance of several compounds, including brain lactate [15], Aβ and tau proteins [11, 16], and biomarkers associated with acute traumatic brain injury [17]. Moreover, strong risk factors associated with AD, such as aging [3], neurovascular injury [18], and sleep disorders [19], are implicated with reduced glymphatic and/or meningeal lymphatic activity [20–23]. On the other hand, therapeutic strategies such as nutritional supplementation [24, 25], non-invasive brain stimulation [26–28], traditional Chinese medicine [29], and physical exercise [30–32] can increase glymphatic and/or meningeal lymphatic activity. Based on that, this review aims at discussing the glymphatic and meningeal lymphatic systems as novel mechanisms involved in AD pathophysiology. Moreover, we explore potential nonpharmacological therapeutic interventions that could reduce such pathophysiological features and improve cognitive performance by increasing glymphatic and/or meningeal lymphatic activity. Finally, the glymphatic system will be discussed as a potential key mechanism underlying the neuroprotective effects of physical exercise in AD.

## The glymphatic and meningeal lymphatic pathways for central solute clearance and their major components

For over 100 years, clinical and experimental scientists have observed tracers injected into the CNS to be apparent a few minutes later in the peripheral lymphatic system [see [33] for an in-depth review and [34] for the discovery of the central lymphatic system]. Such observations suggest a dynamic interaction between the CSF, the interstitial fluid (ISF), and the extracranial lymphatic system [35]. Despite that, tightly regulated vascular permeability and the absence of brain lymphatic vessels pose a challenge to understanding how metabolic waste is cleared from the brain [35].

Rennels and colleagues [12] and Iliff and colleagues [11] pioneered the field demonstrating that bulk CSF flows through the brain parenchyma following a peravascular pathway, clearing interstitial solute and metabolic waste along the way. Since polarized aquaporin 4 (AQP4) water channels on astrocyte endfeet are key to CSF bulk flow, this mechanism was termed the “glymphatic system” given its resemblance with the peripheral lymphatic system and the relevance of glial cells (Iliff et al. 2013; 2012). More recently, two research teams confirmed the existence of meningeal lymphatic vessels by using modern imaging techniques and specific lymphatic markers (Aspelund et al. 2015; Louveau et al. 2015), which were later shown to be functionally connected to the glymphatic pathway, representing a major route for CSF drainage into the cervical lymph nodes (Louveau et al. 2017).

### The glymphatic system

**Fig. 1:**
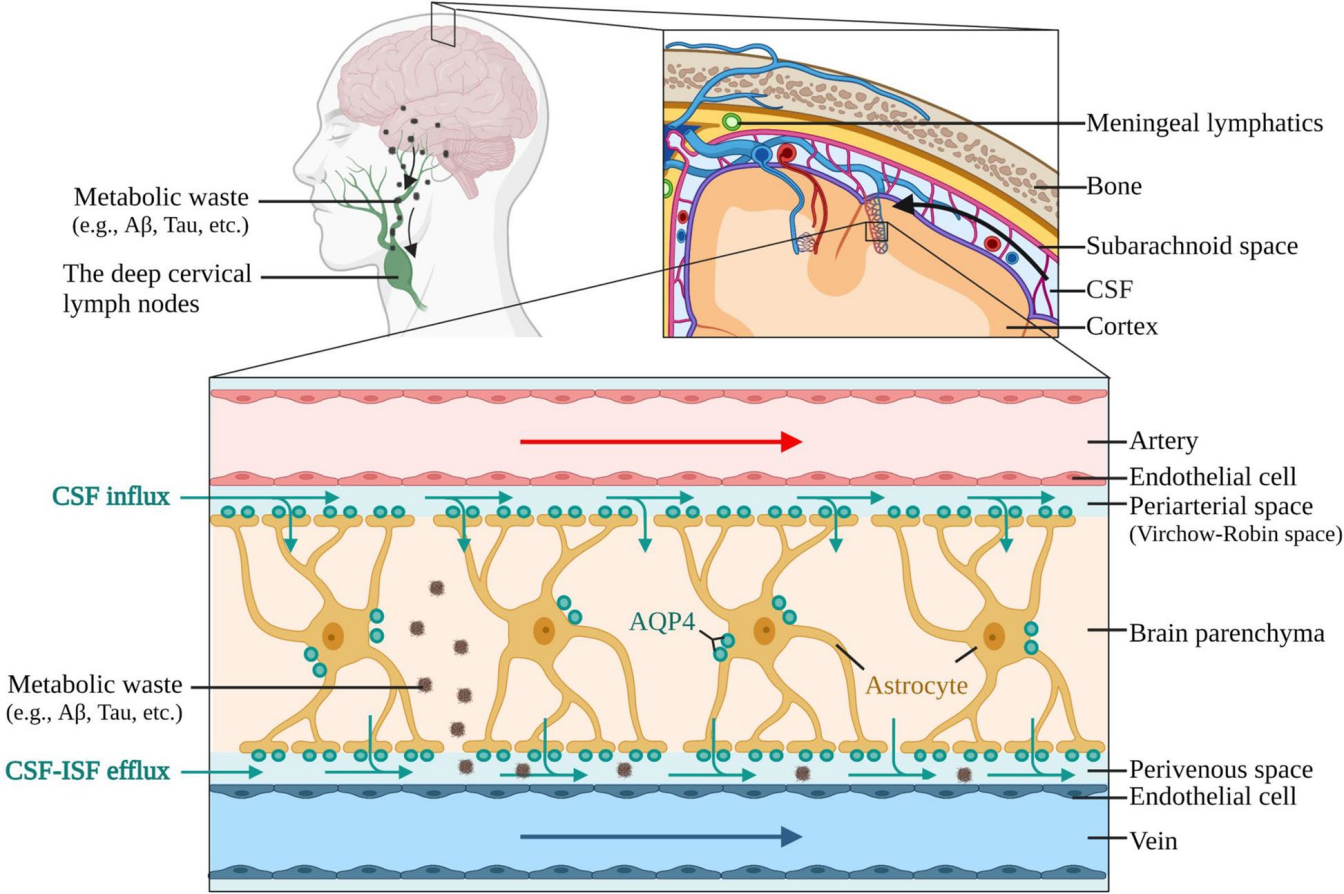
The glymphatic and meningeal lymphatic pathways for central solute clearance and their major components. Cerebrospinal fluid (CSF) enters the brain by moving from the subarachnoid space (superior image) into the space surrounding penetrating arteries (i.e., the periarterial or Virchow-Robin space, as shown in the amplified image), mostly driven by the convective force originated from pulsatile penetrating arteries. From the periarterial space, convective and diffusive forces guide the CSF inflow into the brain parenchyma, aided by polarized aquaporin 4 (AQP4) water channels expressed at astrocyte endfeet. CSF moves along the parenchyma towards the perivenous space, mixing with the interstitial fluid (ISF) and carrying the metabolic waste. The respiration cycle has been suggested as the main force driving CSF-ISF movement through the perivenous space. From the perivenous space, CSF-ISF and the metabolic waste exit the brain mostly through the meningeal lymphatic system (green circles, upper image) that drain into the deep cervical lymph nodes, although outflow along the olfactory, cranial, and spinal nerves into other cervical lymph nodes are also observed

Both ex vivo [11, 12, 36] and in vivo [11, 23, 36, 37] experiments on the CSF-ISF exchange dynamics indicate the glymphatic system is composed of three major anatomical regions: the periarterial space, the brain parenchyma, and the perivenous space (Rasmussen, Mestre, and Nedergaard 2018), while the meningeal lymphatic system is here discussed as one of the main routes for extracranial waste drainage [13, 14]. As depicted in Fig. 1, CSF flows from the subarachnoid space into the periarterial space (also known as Virchow-Robin space), from which it enters the brain parenchyma aided by polarized AQP4 on astrocyte endfeet, mixing with ISF [11]. The kinetics of the CSF glymphatic influx can be as fast as 5 min after intracisterna magna tracer injection in anesthetized animals, suggesting rapid exchange dynamics [37]. Interestingly, CSF glymphatic influx is more pronounced on the ventral brain surface rather than the dorsal [11, 36].

The paravascular space around penetrating arteries can be 1.4 times larger than the vascular cross-sectional area [37] and is interposed in between the other components of the neurovascular unit [38, 39]. On its inner side, the periarterial space is limited by the basal lamina, formed beneath the vascular endothelial cells, and the pericytes covering the vascular endothelium [40, 41], while the outer side is limited by the parenchyma basement membrane adjacent to the pial limitans [38, 42]. Such outer region of the periarterial space is covered to a great extent by astrocyte projections (i.e., astrocyte endfeet) [41]. The passage of CSF from the subarachnoid region to and along the periarterial space is largely the result of convective forces driven by arterial pulsatility [37]. Two-photon imaging paralleled by cardiac cycle recordings shows that the cardiac systolic phase causes an increase in the artery diameter, whereas the diastolic phase decreases it, resulting in CSF being pushed along the Virchow-Robin space through a periarterial pumping system [37]. Congruently, aortic occlusion [12] or internal carotid artery ligation (Iliff et al. 2013) suppresses arterial pulsatility and abrogates CSF tracer influx, whereas pharmacologically increasing heart rate and brain arterial pulsatility significantly increases CSF glymphatic influx [36]. Finally, since vascular pulsatility is observed in arteries but not in veins, this mechanism likely explains why CSF enters the parenchyma through arteries but not veins [36, 37]. Such concept of a “paravascular pump” powering convective transport of solute across the brain parenchyma is not new [43], although its investigation in the context of the glymphatic system has provided clearer experimental evidence [36, 37]. Moreover, Kiviniemi and colleagues [44] demonstrated in humans that the cardiac cycle is coupled with convective CSF movement, especially in the periarterial region. Nonetheless, rodents and humans have important cardiovascular differences [45]. Rodents, for instance, have a basal heart rate that is several times higher than that observed in humans [45]. Given the relevance of heart rate for solute dispersion and CSF paravascular flux, caution should be made on the direct translation of experimental glymphatic findings to the clinical context.

Paravascular CSF tracer intake is largely reduced in the absence of glial AQP4, suggesting CSF bulk flow into the brain parenchyma [11, 37]. AQP4 is particularly expressed in glia and ependymal cells [46] and regulates water transport across the brain [47, 48]. Selective knockout of glial AQP4 results in a 31% reduction in intracerebral water intake after intraperitoneal water loading [49]. Moreover, both the rate of CSF glymphatic influx and ISF solute clearance are reduced by roughly 70% in the absence of APQ4 [11, 37], further supporting its role in regulating CSF bulk flow and glymphatic function. Early immunogold analysis demonstrates that polarized AQP4 expression is highly concentrated in the astrocyte endfeet [46, 50]. Likewise, polarized AQP4 at the astrocyte endfeet rather than the parenchymal membrane is specifically important for glymphatic transport [22]. Although aged and young animals express comparable levels of AQP4, there is a significant reduction in the expression of polarized APQ4 that impairs CSF glymphatic influx [22]. Several lines of evidence demonstrate that AQP4 polarization relies on the pericyte-astrocyte interaction [51–53]. Approximately 40% of the AQP4 expression is restricted to the paravascular region where astrocytes and pericytes interface [51]. Genetic downregulation of the platelet-derived growth factor B (PDGF-B), which is crucial for pericyte recruitment, results in redistribution of AQP4 along the astrocyte membranes and, consequently, loss of AQP4 polarization [52, 53]. On the other hand, astrocyte-derived laminin regulates basal lamina morphology and pericyte differentiation [53, 54], whereas the basal lamina provides the medium for cellular interactions with the extracellular matrix [53]. Such findings suggest that different components of the neurovascular unit could regulate glymphatic function. Indeed, Munk and colleagues [55] demonstrated that the loss of AQP4 polarization due to genetic downregulation of PDGF-B signaling and, consequently, disruption of the pericytes’ coverage leads to impaired glymphatic CSF influx. Although a classical view of the glymphatic pathway accounts mostly for AQP4 polarization, it is conceivable that the neurovascular unit as a whole contributes to glymphatic activity.

Experiments also demonstrate that the brain is still permeable to water in the absence of glial AQP4 [49]. Electron microscopic 3D reconstruction of the gliovascular sheath indicates that, even though the astrocyte endfeet almost completely cover the blood vessels, gaps of around 20 nm are detectable [41]. According to Thrane and colleagues [56], such gliovascular gaps would be large enough to permit the diffusion of water and most mammalian proteins. In agreement with that, injection of tracer in the CSF of AQP4 null mice still results in a gradient fluorescence pattern down to the cortical region that is indicative of tracer transport by diffusion [57]. Such observations led some to question the overall hypothesis of glymphatic transport [58, 59]. In an attempt to replicate the pioneering work on the glymphatic system, Smith and colleagues [58, 59] observed that the rate of CSF tracer influx into the brain parenchyma varies according to the size of the injected particle, suggesting diffusive mechanisms of transport (dispersion) rather than convection (CSF bulk flow). Moreover, they observed that AQP4 knockout did not impair the CSF solute intake or the clearance of fluorescent Aβ injected in the striatal region [58, 59]. Such contradictory findings can be explained by methodological and procedural differences among studies probing the glymphatic system. In a multicenter effort to re-evaluate the relevance of AQP4 for CSF tracer influx, four different AQP4 knockout mouse lines and one mouse line lacking perivascular APQ4 expression were tested [57]. Authors consistently observed reduced solute uptake into the brain parenchyma in ex vivo and in vivo experiments in all the AQP4 null mouse lines [57]. It was also demonstrated that traumatic procedures, such as skull hole drilling or glass pipette insertion in the striatal region (as performed in [58]), result in a brain-wide interruption of glymphatic function [57]. Finally, after meta-analyzing previous research findings, the authors observed that anesthesia, age, and tracer delivery route were the main factors explaining the data variability among studies using APQ4 knockout mouse lines [57]. Indeed, anesthesia can differently affect glymphatic influx, with ketamine and xylazine significantly increasing it and isoflurane suppressing it [60]. Moreover, avertin, the same anesthetic used by Smith and colleagues [58], similarly suppresses glymphatic CSF influx [60], likely explaining the absence of glymphatic transport observed in their investigation. Based on such findings, although unlikely to be the only form of CSF and solute transport across the brain, AQP4-dependent convection (i.e., the glymphatic pathway) appears to be a major route.

According to the glymphatic pathway depicted in Fig. 1, after leaving the periarterial space the CSF moves along the brain parenchyma towards the perivenous space, from where CSF-ISF exits the parenchyma [11, 12, 36]. As for glymphatic influx, glial AQP4 has been proposed as the substrate for perivenous glymphatic efflux [56, 61]. In support of this hypothesis, AQP4 expression has also been implicated in water resorption associated with vasogenic edema [47, 62]. Six hours after focal cortical freeze injury, AQP4 knockout mice present increased brain water content, greater intracranial pressure, and worse neurological outcome compared to their wild-type counterparts [62]. Moreover, such knockout animals have impaired brain water drainage upon continuous (60 min) cerebral infusion of an isotonic solution [62]. Moreover, around 10% of AQP4 knockout animals develop hydrocephalus within 2–3 weeks after birth [63], which is about the same age others have noted a small but significant increase in brain water content in AQP4 null rodents [49]. Although such findings point towards the involvement of AQP4 in brain water resorption under pathological and physiological conditions, direct experimental evidence for the AQP4 contribution to glymphatic CSF-ISF efflux warrants further investigation.

Cardiac pulsatility has been established as the main force driving periarterial CSF flow, although the relevance of the respiratory cycle for CSF movement has also been investigated [64]. As observed by ultra-fast magnetic resonance encephalography in healthy subjects, cardiac pulsation (~ 1 Hz) is a source of convective movements mainly in the periarterial region, whereas respiration (~ 0.3 Hz) appears to exert a convective role in the perivenous region [44]. Based on that, the cardiac and respiratory cycles may contribute to different aspects of the glymphatic pathway, with arterial pulsatility driving the periarterial CSF inflow and respiration driving the perivenous CSF-ISF outflow.

A remarkable discovery is that the glymphatic system is almost exclusively active during sleep [23], which hints at the evolutionary relevance of sleeping. During sleep, there is a ~ 60% increase in the interstitial space volume, resulting in reduced convective resistance in association with a ~ 95% increase in CSF glymphatic influx [23]. Likewise, pharmacological inhibition of the central adrenergic system in awake mice, which mimics the sleep-associated increase in interstitial space volume, leads to an increase in CSF glymphatic influx [23]. Moreover, ISF solute clearance is two times faster during sleep [23], whereas sleep deprivation largely reduces glymphatic activity [17, 65].

The dynamics of CSF production and the glymphatic system share some intriguing similarities. CSF production follows a circadian rhythm, peaking during the night [66] when the glymphatic system is most active [23]. Such circadian production of CSF reflects the recently described function of the choroid plexus as a circadian clock [67]. Likewise, the relevance of CSF production on glymphatic activity has also been investigated [17]. Plog and colleagues [17] demonstrated that the pharmacological reduction of CSF production through acetazolamide (a carbonic anhydrase inhibitor) results in a significant decrease in ISF tracer clearance. However, since solute clearance was measured by the strength of the tracer signal in the dcLN [17], such findings could exclude the influence of the meningeal lymphatics in the reduced tracer efflux. The relevance of CSF production on glymphatic activity was also questioned based on the opposing effects that isoflurane has on the two systems [60, 68]. Isoflurane significantly increases CSF production compared with other anesthetic compounds [68] while largely suppressing glymphatic function [60]. Such findings indirectly suggest that CSF production and glymphatic activity follow distinct regulatory mechanisms, although such hypothesis requires further confirmatory experiments. Furthermore, such experiments should consider the great array of techniques for measuring CSF production, their limitations, and the differences between rodents and humans (see [68] for an in-depth review on this topic).

Finally, the CSF-ISF that egresses from the glymphatic pathway can be drained out of the skull through an extensive network of meningeal lymphatic vessels that were recently described with the aid of modern imaging techniques [13, 14].

### The meningeal lymphatic system

The meningeal lymphatic system is formed by a network of lymphatic vessels located in the dura mater, extending along the perisinusoidal spaces and exiting the cranium alongside veins, cranial nerves, and arteries foramina at the base of the skull [13, 14, 20, 69]. It develops in the first four weeks after the rodent’s birth [70], relying on the vascular endothelial growth factor (VEGF) signaling, especially the VEGF-C/VEGFR3 signaling pathway [13, 20, 70]. Knockout of VEGFR3 results in severe disruption of the meningeal lymphatics development [13, 70], whereas its downregulation in adult mice leads to meningeal lymphatic regression [20, 70]. Remarkably, some lymphatic re-growth is observed after restoring the VEGFR3 expression in adult mice, suggesting such system retains certain plasticity throughout adult life [70]. More recently, the Down syndrome critical region 1 (DSCR1) protein, which is upregulated by VEGF during the initial period of lymphangiogenesis, was shown to increase meningeal lymphatic branching in adult mice, especially on the dorsal region [71].

The meningeal lymphatic system has been implicated in brain waste clearance [13, 14, 20, 21, 71]. Although lymphatic vessels are observed on the dorsal and basal surfaces [13, 20], they have different morphological characteristics [20]. The meningeal lymphatics on the base of the skull (along petrosquamosal sinus and sigmoid sinus) depict several blunt protrusions with oak leaf-shaped lymphatic endothelial cells joined by button-like junctions [20] that are morphologically similar to the peripheral initial lymphatics [72]. Moreover, such initial-like lymphatics connect into vessels harboring lymphatic valves and mixed button- and zipper-like junctions [20], which are characteristics of pre-collector lymphatic vessels. Dorsal meningeal lymphatics, on the other hand, are discontinuous, have a smaller diameter, and have no lymphatic valves [14, 20]. Although button-like junctions were observed along such dorsal vessels [14, 20, 73], they were less abundant and morphologically different from those observed in functional peripheral lymphatics [20, 72, 73].

Such morphological discrepancies may underlie the differences observed in dorsal and basal lymphatic drainage. Intracerebroventricular infused tracer is firstly detected by MRI in the basal outflow region and at a much later stage in the dorsal region, suggesting the basal meningeal lymphatics drain CSF more efficiently than the dorsal lymphatics [20]. Some failed to detect CSF-injected tracers in the dorsal region [20, 74], although it was previously demonstrated by multiphoton microscopy imaging [14]. This could be due to the fact that CSF drainage at the dorsal region occurs preferably in the lymphatics along the transverse sinus and in those around the olfactory bulb (designated as “hotspots”), but not in the lymphatics adjacent to the middle meningeal artery or the superior sagittal sinus [73]. In agreement with that, selective ablation of the dorsal meningeal lymphatics results in reduced tracer drainage into the cervical lymph nodes [21, 73, 75], whereas increasing dorsal meningeal lymphatic branching by genetic overexpression of the DSCR1 increases it [71]. Finally, by using postmortem light sheet fluorescence microscopy, Jacob and colleagues [69] confirmed CSF tracer uptake in both dorsal and basal hotspots by reconstructing a three-dimensional representation of CSF-tracer distribution in the entire mouse head. The authors also identified three additional cranial sites of CSF lymphatic drainage, the cavernous (caudal and rostral region), intraorbital, and nasal lymphatic vessels [69]. Such findings indicate that both dorsal and basal meningeal lymphatics are relevant for CSF drainage.

Evidence also suggests the meningeal lymphatic system is an important exiting route for glymphatic-cleared waste. ISF-injected tracers are observed in the basal meningeal lymphatics and cervical lymph nodes [13, 20], while ligation of the dcLN afferents results in tracer accumulation in the meningeal lymphatics [13]. Likewise, selective disruption of dorsal meningeal lymphatics leads to impaired ISF tracer efflux to the dcLN [21]. Remarkably, Da Mesquita and colleagues [21] demonstrated that disruption of such system also impairs CSF glymphatic influx (in the absence of observable expression changes in polarized AQP4), suggesting the meningeal lymphatic and glymphatic systems are functionally connected.

Apart from brain waste clearance, the meningeal lymphatics also participate in CNS immunosurveillance [73]. Through experiments using photoconversion of fluorescent proteins, Louveau and colleagues [73] demonstrated that meningeal T cells egress to cervical lymph nodes through the meningeal lymphatics, which depends on the C–C chemokine receptor type 7 (CCR7) signaling. In agreement with that, downregulation of CCR7 signaling results in meningeal T cell accumulation [73, 76]. Moreover, in an animal model of neuroinflammation, meningeal lymphatic ablation or ligation of the dcLN reduces the neuroinflammatory response and the disease severity [73], suggesting neuroimmune regulation is largely dependent on T cell egress to the cervical lymph nodes through the meningeal lymphatics.

Such complex lymphatic system has been observed in humans both through contrast-enhanced MRI [69, 77–79] and immunohistochemical analysis of postmortem tissue samples [80, 81]. Using contrast-enhanced MRIs at multiple time points, Zhou and colleagues [78] demonstrated that agent clearance through the glymphatic and meningeal lymphatic pathways is highly correlated, with earlier signal detection in the meningeal lymphatics associated with faster glymphatic clearance. Given that contrast-enhanced MRI might not be suitable for healthy individuals, Albayram and colleagues [82] recently devised a new 3D fluid-attenuated inversion recovery (FLAIR) magnetic resonance method that requires no contrast and permits the visualization of the dorsal and basal meningeal lymphatics and their link with the dcLN. Of note, such authors observed that the meningeal lymphatics at the dorsal region were as prominent as the basal meningeal lymphatics [82], contradicting previous observations in rodents [20]. Such difference could be due to a significantly more developed telencephalon in humans, which would demand an equally larger dorsal lymphatic coverage [82].

One aspect that remains to be demonstrated is the mechanism by which leptomeningeal CSF can pass through the arachnoid barrier and reach the lymphatics present in the dura mater. Although the mechanism remains unknown, Ahn and colleagues [20] observed that initial lymphatics at the basal region of the brain are positioned closer to the subarachnoid space, suggesting a privileged spatial orientation for basal lymphatics to access the leptomeningeal CSF. Nonetheless, the relevance of the meningeal lymphatic system for central immunosurveillance and brain waste clearance has been consistently demonstrated. Moreover, a functional connection between the glymphatic and meningeal lymphatic systems suggests they can be involved in the etiology of proteinopathies. Since the extracellular accumulation of toxic proteins is a hallmark of AD [5–7], it is reasonable to hypothesize that the disruption of such systems may contribute to AD pathogenesis.

## The glymphatic and meningeal lymphatic systems in the pathophysiology of Alzheimer’s disease

Aging is the strongest risk factor associated with AD [3]. The prevalence of AD and other types of dementia doubles every 5.5 years after 60 years of age [3]. Nonetheless, the relationship between aging and neurodegeneration is not completely understood. The predictive value of Aβ accumulation for AD development decreases with aging and is no longer associated with the disease at the age of 95 years [83]. It indicates that premature accumulation of extracellular protein is a disease biomarker rather than extracellular protein accumulation per se*,* suggesting other factors, such as regulatory clearance mechanisms, may precede the proteinopathy.

**Fig. 2:**
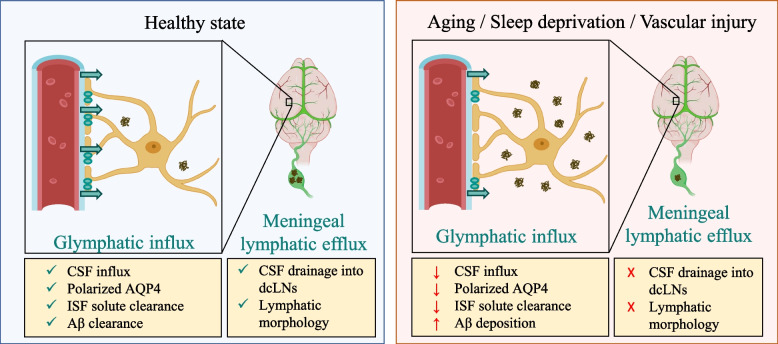
The glymphatic and meningeal lymphatic pathways for brain waste clearance in different health conditions. In healthy adult rodents (left panel), CSF glymphatic influx is facilitated by polarized AQP4 richly expressed at the astrocyte endfeet, while the flow of CSF-ISF along the glymphatic pathway clears the parenchyma solute (including Aβ), which is then drained into the cervical lymph nodes by the extensive meningeal lymphatic vasculature on the dorsal and basal regions of the brain. On the other hand, aging, sleep deprivation, and vascular injury (right panel) are associated with impairments in the glymphatic function, resulting in reduced CSF glymphatic influx and ISF solute clearance. Moreover, aging is also associated with morphological and functional alterations in the meningeal lymphatics that result in reduced lymphatic drainage into the deep cervical lymph nodes (CSF, cerebrospinal fluid; AQP4, aquaporin 4; ISF, interstitial fluid; Aβ, amyloid beta; dcLN, deep cervical lymph nodes)

As depicted in Fig. 2, both the glymphatic and meningeal lymphatic systems are affected by aging. Middle-aged rodents (10–12 months) have reduced arterial pulsatility and reduced expression of polarized AQP4, leading to impaired CSF glymphatic influx and ISF tracer efflux [22]. Aging is also accompanied by hyperplasia of the basal meningeal lymphatics and a reduction in button-like junctions [20], while the dorsal lymphatics tend to retract and reduce their coverage [20, 21, 70]. Similarly, contrast-enhanced MRI suggests that both the glymphatic and meningeal lymphatic functions are reduced by aging in humans [78, 79]. Such senescence of the central clearance systems potentially underlies the aging-associated cognitive decline, as cognitive performance is positively correlated with the clearance function of putative meningeal lymphatics observed by contrast-enhanced MRI [79]. Likewise, knockout of AQP4 [84] or chronic impairment of the meningeal lymphatics in rodents [21] impairs spatial learning and memory. Moreover, aging is also associated with meningeal lymphatics immunosenescence, as decreased expression of CCR7 and increasing accumulation of meningeal T cells are observed in aged mice [76]. Remarkably, CCR7 knockout leads to a significant reduction in the expression of polarized AQP4 and impaired CSF glymphatic influx in adult mice [76], suggesting the senescence of one system can affect the other.

Many aging-associated impairments in the glymphatic and meningeal lymphatic function are also observed in AD. Patients with AD have increased AQP4 immunoreactivity but markedly reduced expression of polarized AQP4 when compared with age-matched healthy controls (as observed by postmortem analysis of tissue samples) [85], confirming similar observations in animal models of AD [84]. Moreover, the severity of vascular Aβ deposition in AD patients (i.e., Aβ angiopathy) is associated with lower expression of perivascular AQP4 [86], suggesting a link between impaired glymphatic function and toxic protein accumulation. Indeed, increased perivascular diffusivity, a proposed marker for measuring glymphatic function through MRI, is positively associated with cognitive performance in AD patients [87]. Curiously, *postmortem* meningeal lymphatic samples of humans with AD did not present Aβ accumulation or reduced vessel diameter compared with age-matched controls [80]. Similarly, reduced coverage of dorsal meningeal lymphatics in a familial animal model of AD is observed as a function of aging rather than the genetic modeling [75], as reduced lymphatic coverage and drainage function is observed in old but not adult AD mice [21, 75]. Nonetheless, pharmacological ablation of the dorsal meningeal lymphatics leads to early accumulation of meningeal Aβ and increased hippocampal Aβ burden in different AD animal models [21]. Moreover, CCR7 knockout (which regulates the meningeal lymphatic immune function) leads to increased AD-related pathology (increased Aβ deposition, brain vascular damage, and microglial activation) and worse cognitive profile in a familial animal model of AD [75]. Such findings point towards a link between impaired central waste clearance and toxic protein accumulation, although the involvement of the meningeal lymphatics in the early experimental stages of the disease requires further investigation.

Another important factor associated with aging is the disruption of sleep patterns and a higher incidence of sleep disorders [88, 89]. Decreased sleep duration and quality, increase in sleep fragmentation, greater difficulty falling asleep, and less rapid eye movement sleep are common sleep disturbances observed in the elderly [88]. As observed in a large populational study, sleep disturbances are strongly linked with faster cognitive decline [90] and the development of dementia [91]. Indeed, more than 50% of AD patients are affected by sleeping disorders [92]. In connection with that, both clinical and experimental findings demonstrate that the extracellular levels of AD-associated proteins are modulated by sleep [93, 94]. In rodents, ISF Aβ levels are 25% lower during the dark phase [94], while tau levels are 90% lower during resting compared with the animals’ active phase [93]. A similar pattern is observed in humans [93, 94]. Not surprisingly, sleep deprivation increases extracellular protein accumulation in humans and in experimental models of AD [93, 94]. In healthy humans, acute sleep deprivation increases by 30% and by 50% the CSF levels of Aβ and tau protein [93], respectively, while increasing Aβ burden in the hippocampal formation and thalamic region (as measured by positron emission tomography [PET]) [95]. Moreover, it is estimated that cumulative sleep deprivation explains 25% of the central Aβ burden [95]. Indeed, as observed in animal models of AD, chronic sleep deprivation (21 days) leads to increased central Aβ deposition [94]. Given that the glymphatic system is most active during sleep [23] and that sleep deprivation significantly reduces glymphatic function [17], such findings suggest aging and AD-associated sleep disorders could further contribute to toxic protein accumulation in the brain by affecting the glymphatic system. However, the influence of the sleep–wake cycle or a circadian rhythm on the meningeal lymphatic function is not yet clear.

Finally, the glymphatic system can be integrated into the two-hit vascular hypothesis of AD. According to this hypothesis, Aβ-independent mechanisms would lead to clinically silent vascular deterioration and impaired Aβ clearance (hit 1), resulting in the increased Aβ accumulation (hit 2) that accompanies the clinical expression of AD [96, 97]. Indeed, vascular injury is strongly correlated with aging and precedes any observable cognitive deficits in both humans and rodents [98, 99]. BBB integrity is largely maintained by the components of the neurovascular unit, including astrocytes, pericytes, and basal lamina [40, 100], such that disruption of these components leads to BBB breakdown and increased vascular injury [18, 96, 98, 99]. As previously discussed, the neurovascular unit also regulates the glymphatic function [55], suggesting that neurovascular deterioration would result in mutual impairment of the vascular and glymphatic functions. Nonetheless, such integrative perspective lacks direct experimental evidence, and a cause-effect relationship between vascular integrity and glymphatic function in AD pathogenesis warrants further exploration.

As summarized in Fig. 2, impairment to the glymphatic and meningeal lymphatic systems due to aging, sleep deprivation, or vascular injury can contribute to AD pathogenesis. The relevance of such clearance pathways has increased the interest in central fluid dynamics as a potential therapeutic strategy for several neurodegenerative disorders [8]. In agreement with the two-hit vascular hypothesis of AD, targeting such clearance systems that are potentially involved in the Aβ-independent hit 1 could be an effective treatment strategy to reduce AD proteinopathy (hit 2).

### The glymphatic and meningeal lymphatic systems as therapeutic targets in Alzheimer’s disease

The treatment of AD involves a combination of nonpharmacological and pharmacological interventions. Psychoeducation and support to the patient and family are usually followed by the prescription of a limited number of cognitive-enhancing agents [5]. The cholinergic and glutamatergic hypothesis of cognitive decline led to the development of cholinesterase inhibitors and the N-methyl-D-aspartate (NMDA) antagonist, memantine [101, 102]. More recently, the Aβ hypothesis of AD has fostered the development of anti-amyloid agents [103–105], leading to the FDA approval of the monoclonal antibody Aducanumab [106] and, more recently, the Lecanemab [107], while other antibody-based interventions, such as the Gantenerumab [108], are likely to be approved soon [104, 105]. However, such anti-Aβ treatments are questioned due to limited improvements in the patient’s cognitive function and possibly fatal side effects [105, 109]. Therefore, the development of an effective treatment that halts disease progression remains remarkably challenging.

The recent findings here discussed bring forward the possibility of counteracting disease progression by enhancing the brain’s endogenous waste clearance pathways. Given the burden of polypharmacy among the elderly [110] and the relevance of lifestyle factors for healthy aging [96, 111], we explore below the experimental evidence associating nonpharmacological interventions with improved glymphatic and/or meningeal lymphatic function in animal models of AD (summarized in Table 1). However, it is important to mention that such interventions, like physical exercise and nutrition, have very broad effects that extend beyond their potential use as brain waste clearance enhancers [112, 113]. Narrowing down their therapeutic potential to a single mechanism, therefore, is beyond our expectations. Instead, we review the experimental evidence implicating the glymphatic and meningeal lymphatic systems as newly described contributive factors.

**Table 1:** Nonpharmacological effects on glymphatic and meningeal lymphatic function in animal models of AD

Ref	Intervention	Animal model	Glymphatic and meningeal lymphatic effects	Neurophysiological outcomes	Brain Structures	Behavioral outcomes
Ren et al. (2017) [24]	↑ PUFAs *(Fat-1* mice or fish oil supplementation)	Aβ (i.c.v.)	↑ CSF influx ↑ ISF solute clearance	↓ Aβ deposition ↓ Neuronal loss	Hippocampus	↑ Learning and memory
Zinchenko et al. (2019) [27]	tPBM	Aβ (i.c.v.)	↑ CSF drainage into dcLNs	↓ Aβ deposition	Hippocampus and cortex	Not investigated
Semyachkina-Glushkovskaya et al. (2021) [28]	tPBM	Aβ (i.c.v.)	↑ CSF drainage into dcLNs	↓ Aβ deposition	Not specified	↑ Learning and memory
Lee et al. (2020) [26]	FUS-MB	5xFAD mice	↑ CSF drainage into dcLNs	↓ Aβ deposition ↓ Gliosis	Entorhinal cortex and hippocampus	Not investigated
Lin et al. (2021) [114]	TMS	5xFAD mice	↑ CSF influx ↑ CSF drainage into dcLNs	↓ Aβ deposition ↓ Gliosis ↑ Neuronal activation	mPFC and hippocampus	↑ Learning and memory
Liang et al. (2021) [115]	Electroacupuncture	SAMP8 mice	↑ Polarized AQP4 ↑ CSF influx	↓ Neuronal loss ↓ Aβ deposition	Hippocampus and cortex	↑ Learning and memory
Lyu et al. (2021) [116]	Yi-Zhi-Fang-Dai	Stroke (ischemia–reperfusion)	↑ Polarized AQP4	↓ Aβ deposition	Hippocampus and cortex	Not investigated
Zhang et al. (2021) [25]	NBP	APP/PS1 mice	↑ Polarized AQP4	↓ Aβ deposition	Hippocampus and cortex	↑ Learning and memory
Zheng et al. (2021) [117]	Xueshuantong	APP/PS1 mice	↑ Meningeal lymphatics morphology ↔ AQP4 Expression	↓ Aβ deposition ↓ Inflammation	Meninges	Not investigated

↑ increased/improved; ↓ reduced/decreased; ↔ unchanged. *PUFAs* n-3 polyunsaturated fatty acids, *tPBM* Transcranial photobiomodulation, *FUS-MB* Focused ultrasound therapy combined with intravenous injection of microtubules, *TMS* Transcranial magnetic stimulation, *NBP* L-3-n-butylphthalide

Nutritional supplementation with different compounds has been well-regarded as a strategy to prevent and manage dementia [118]. Higher quantities of n-3polyunsaturated fatty acids (PUFAs), both endogenous (as observed in *Fat-1* mice) or exogenous (following 3 weeks of fish oil supplementation), protect against intracerebroventricular Aβ infusion, significantly reducing hippocampal Aβ deposition, neuronal loss, and improving spatial learning and memory [24]. Remarkably, the authors reported that higher levels of PUFA increased CSF-ISF exchange dynamics, as indicated by faster CSF glymphatic influx and ISF solute clearance when compared to controls [24]. Moreover, oral administration of L-3-n-butylphthalide (NBP), a compound derived from celery oil, acutely [2 h] increases the pulsatility of surface and penetrating brain arteries, enhancing CSF-ISF exchange dynamics as observed by increased CSF glymphatic influx, ISF solute clearance, and increased meningeal lymphatic outflow into the dcLNs [25]. NBP treatment for three months increases polarized AQP4 expression and reduces Aβ plaque formation in the hippocampus and cortex, which leads to increased spatial learning and memory in transgenic AD animals [25].

Noninvasive brain stimulation is another potential strategy for AD management [119]. In an Aβ-induced animal model, nine days of transcranial photobiomodulation (tPBM) reduces cortical and hippocampal Aβ deposition [27] and improves cognitive performance [28]. Of note, tPBM performed during the rodents’ dark phase clears Aβ two times faster than tPBM during the light phase [28]. Likewise, increased glymphatic and meningeal lymphatic function is being suggested as a potential mechanism of action. tPBM increases up to 9 times the rate of ISF solute clearance compared to control animals [27]. Similarly, it also increases meningeal lymphatic outflow into the dcLNs [28]. Furthermore, six sections of focused ultrasound therapy, an alternative noninvasive brain stimulation technique, combined with intravenous injection of microtubules (FUS-MB), increases Aβ clearance into the dcLNs and reduces reactive gliosis in the hippocampus and entorhinal cortex in an animal model of AD [26].

Repetitive transcranial magnetic stimulation (TMS) has been effective in counteracting AD proteinopathy and improving cognitive function in the early stages of the disease [114, 120]. In a transgenic animal model, 14 days of TMS reduces Aβ deposition in the hippocampus and mPFC and prevents spatial memory loss [114]. Remarkably, such neuroprotective effects are associated with increased CSF-ISF exchange dynamics, as observed by increased CSF glymphatic influx and meningeal lymphatic outflow into the dcLNs, resulting in reduced gliosis and increased neuronal activation in the hippocampus and mPFC [114]. Of note, a single session of TMS using continuous theta burst stimulation can restore the sleep deprivation-induced reduction in the expression of polarized AQP4 [65] and increase meningeal lymphatics dilation [121], suggesting TMS increases clearance function by acting on both the glymphatic and meningeal lymphatic pathways.

Traditional Chinese medicine has a history of more than two thousand years of treating and managing dementia [122, 123] and recent evidence suggests it can also increase glymphatic and meningeal lymphatic activity (see [29] for a recent in-depth review). Eight weeks of electroacupuncture increases perivascular AQP4 expression and CSF glymphatic influx in 7 months-old rats with accelerated senescence (SAMP8 mice), resulting in reduced hippocampal and cortical Aβ deposition and improved learning and memory when compared to untreated animals [115]. The Chinese herbal medicine formula of Yi-Zhi-Fang-Dai protects against ischemic stroke-induced downregulation of perivascular AQP4 and the resulting increase in cortical and hippocampal Aβ deposition, suggesting the possible involvement of the glymphatic pathway in its neuroprotective effects [116]. Moreover, Xueshuantong, another Chinese medicine herbal formula, partially reverses meningeal lymphatics morphology in association with reduced meningeal Aβ accumulation and central pro-inflammatory response in transgenic AD animals [117].

In addition, the meningeal lymphatic system has also been explored as a therapeutic target. The VEGF-C and DSCR1 proteins are involved with lymphangiogenesis and were proposed as potential targets to increase the activity of the meningeal lymphatics in animal models of AD [21, 71]. Transgenic overexpression of the DSCR1 protein leads to increased branching of the dorsal meningeal lymphatics [71]. Such morphological changes are associated with increased ISF solute clearance and meningeal lymphatic outflow into the dcLNs [71]. In AD animals, such effects are associated with a 50% reduction in hippocampal Aβ deposition, absent hippocampal neuronal death, and improved spatial learning and memory performance [71]. Increasing VEGF-C expression by adenoviral gene therapy increases lymphatic vessel diameter in both young and aged mice, which is associated with increased CSF drainage into the dcLNs [21]. Moreover, it increases learning and memory performance in aged animals [21]. However, although VEGF-C overexpression can recover morphological and functional aspects of the meningeal lymphatics in aged mice, it had no significant therapeutic effects in two different transgenic animal models of AD using adult mice [21]. As mentioned earlier, adult AD mice (5-6 months old) are not afflicted by meningeal lymphatic impairments until they get older (> 13 months old) [75], which might explain why VEGF-C adenoviral therapy had no observable effect in the AD animal models using adult mice.

In summary, the emerging roles of the glymphatic and meningeal lymphatic systems in the pathophysiology of AD provide new mechanistic insights for nonpharmacological interventions and potentially novel therapeutic strategies to tackle proteinopathy and disease progression. However, many interventions are still in their experimental phase, have side effects, or can be costly to the patients. Physical exercise, on the other hand, is one of the most well-characterized pro-cognitive interventions [124, 125], which has been highlighted as a therapeutic strategy in AD [112, 118, 126, 127]. It can increase the clearance of extracellular protein in AD animal models [126, 128], although the mechanisms of action remain largely unknown.

## Physical exercise improves glymphatic function in Alzheimer’s disease

Over a third of AD cases are due to preventable risk factors, with physical inactivity accounting for 12.7% of such proportion [111]. There is a well-established relationship between higher levels of physical activity and reduced risk of cognitive decline and dementia [129–131]. In a 10-year follow-up study, higher levels of physical activity were negatively associated with cognitive impairment onset [129]. Moreover, a recent meta-analysis estimated that physical activity is especially protective against AD compared to other forms of dementia, reducing the risk of disease development by 38% [131]. There is strong experimental evidence indicating that the degree of cognitive protection is associated with a reduction in extracellular protein deposition [126, 128]. Moreover, clinical investigations also offer evidence of improved proteinopathy [126]. In this regard, recent experimental evidence suggests a link between physical exercise and increased glymphatic function in both healthy animals and AD animal models.

As summarized in Table 2, voluntary wheel running for 6 to 8 weeks increases the CSF-ISF exchange dynamics in healthy middle-aged animals (7-8 months old) and in an animal model of AD [30, 32]. In healthy middle-aged animals, aerobic exercise increases CSF glymphatic influx and meningeal lymphatic outflow into the dcLNs, and enhances ISF solute clearance, resulting in reduced extracellular Aβ deposition, astrogliosis, increased structural and functional plasticity, and better cognitive performance when compared with age-matched sedentary controls [30]. Interestingly, in 2-month-old healthy animals, physical exercise can increase the rate of CSF glymphatic influx during the light cycle only (when the glymphatic system is barely active), which is not associated with increased polarized AQP4 expression or ISF solute clearance [31]. Nonetheless, it is remarkable that lifestyle factors can still modulate glymphatic function in young adult mice, while such modulation has potentially broader effects and a functional impact only in aged mice.

**Table 2:** Physical exercise effects on glymphatic and meningeal lymphatic function

Ref	Exercise protocol	Animals	Glymphatic and meningeal lymphatic effects	Neurophysiological Outcomes	Brain Structures	Behavioral outcomes
He et al. (2017) [30]	Voluntary wheel running (6 weeks)	Old mice (7–8 months old)	↑ Polarized AQP4 ↑ CSF influx ↑ ISF solute clearance ↑ CSF drainage into dcLNs	↓ Reactive gliosis ↓ Aβ deposition ↑ Dendritic spines ↑ Synaptic proteins	Hippocampus and cortex	↑ Learning and memory
Liu et al. (2022) [32]	Voluntary wheel running (8 weeks)	5 months APP/PS1	↑ Polarized AQP4 ↑ CSF influx	↓ Aβ deposition ↑ BDNF ↑ Synaptic proteins ↓ Reactive gliosis ↓ Neuroinflammation	Forebrain	↑ Learning and memory
		9 months APP/PS1	↔ Polarized AQP4 ↔ CSF influx	↔ Aβ deposition ↔ BDNF ↔ Synaptic proteins ↔ Reactive gliosis ↔ Neuroinflammation	Forebrain	↔ Learning and memory
von Holstein-Rathlou et al. (2018) [31]	Voluntary wheel running (5 weeks)	Young adult mice (2 months old)	↑ Awake CSF influx ↔ CSF influx during sleep ↔ ISF solute clearance ↔ Polarized AQP4	↔ Reactive gliosis	Hippocampus and cortex	Not investigated

↑ increased/improved; ↓ reduced/decreased; ↔ unchanged

When investigated in an animal model of AD overexpressing Aβ (APP/PS1), adult mice (5 months old) that have access to running wheels have increased glymphatic influx and reduced levels of extracellular Aβ and neuroinflammation, resulting in increased plasticity and cognitive performance compared to sedentary animals [32]. Noteworthy, enhancement of the glymphatic function by physical exercise is associated with increased expression of polarized AQP4 [32]. As demonstrated by Liu and colleagues [32], AQP4 knockout in APP/PS1 transgenic animals abrogates the improvements in spatial learning and memory by voluntary wheel running. Moreover, AQP4 deficiency diminishes the effects of running in decreasing Aβ deposition and increasing BDNF signaling and synaptic protein expression in the forebrain [32]. Therefore, at least in adult animals at the early stage of AD transgenic models, physical exercise appears to increase glymphatic activity by reverting the disease-associated reduction in polarized AQP4 expression.

In this regard, the glymphatic system can be a potential key mechanism mediating the preventive and therapeutic effects of physical exercise in dementia [30, 32]. Clinical evidence supports the notion that physical exercise is effective in the early but not the late stage of AD [132]. Consistently, Liu and colleagues [32] reported that voluntary wheel running reduces astrogliosis and increases AQP4 polarization in younger (5 months old), but not older (9 months old) APP/PS1 transgenic animals. Remarkably, at this age, the aerobic exercise protocol does not affect CSF glymphatic influx nor does it improve the neuropathological and cognitive impairments associated with the disease [32]. Further investigations are needed to identify which factors potentially hinder the therapeutic effects of physical exercise at the later stages of the disease. Furthermore, although physical exercise enhances meningeal lymphatic outflow into the dcLN in middle-aged mice, direct effects on the meningeal lymphatic morphology have not been investigated so far. Moreover, the interaction between physical exercise and the meningeal lymphatic system in animal models of AD remains unexplored.

## Conclusion and future perspectives

**Fig. 3:**
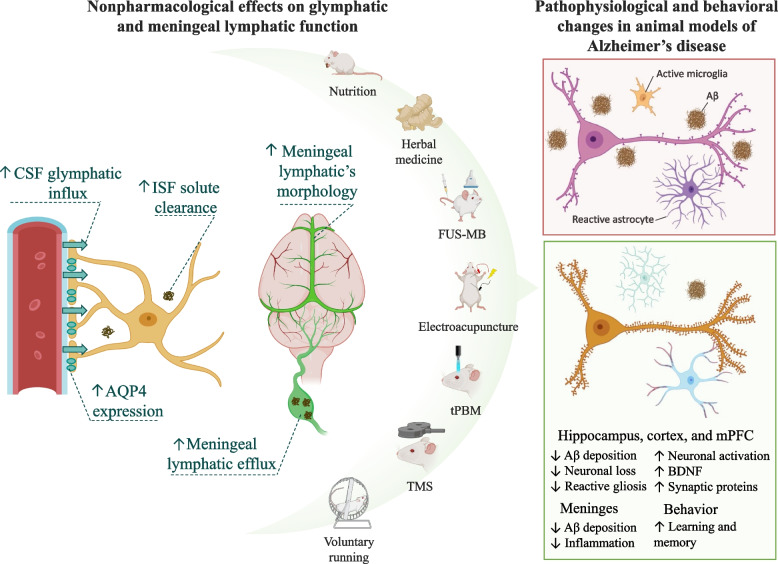
Nonpharmacological effects on the glymphatic and meningeal lymphatic systems in preclinical studies and associated improvements in the disease pathophysiology and behavioral deficits in animal models of AD. Physical exercise (voluntary wheel running), noninvasive brain stimulation (TMS, tPBM, FUS-MB), traditional Chinese medicine (electroacupuncture and Chinese herbal medicine formula Yi-Zhi-Fang-Dai and Xueshuantong), and nutritional supplementation (PUFAs and NBP) have been shown to modulate many factors associated with the glymphatic and meningeal lymphatic systems under physiological conditions (left panel), including the expression of polarized AQP4, CSF glymphatic influx, ISF solute clearance, and the meningeal lymphatics morphology. In animal models of AD, the increased glymphatic and meningeal lymphatic function induced by such nonpharmacological interventions are associated with improvements in the disease pathophysiology (right panel), including reduced Aβ deposition, reactive gliosis, and neuronal loss that culminate with improved learning and memory

AD is a debilitating condition associated with a progressive accumulation of toxic proteins and neurodegeneration. Although our understanding of the disease course has increased appreciably during the last decades, the development of disease-modifying therapeutics remains a challenge. Recent findings attesting to the relevance of the glymphatic and meningeal lymphatic systems for brain waste clearance and immunosurveillance could help overcome these limitations. As here reviewed, aging-associated sleep disorders, vascular injury, reduced polarized AQP4 expression, and morphological alterations in the meningeal lymphatics coherently connect risk factors with the disease-associated proteinopathy (Fig. 2). Likewise, as summarized in Fig. 3, the upregulation of these two systems has been demonstrated as a mechanism underlying the improvements induced by many nonpharmacological interventions on the neuropathological and behavioral deficits observed in animal models of AD. Nonetheless, targeting the glymphatic and meningeal lymphatic systems as potential therapeutic strategies for preventing and/or treating AD is still in its infancy, with many research questions to be answered and targeting strategies to be explored.

Brain solute and metabolic waste clearance are key features associated with the glymphatic system. Nonetheless, this pathway can also be involved in the delivery of nutrients and other factors to the brain. It has been demonstrated that apolipoprotein E (ApoE), a key regulator of the transport and metabolism of cholesterol and a risk factor for AD, is delivered from CSF to the brain parenchyma through the glymphatic pathway [133]. Moreover, the glymphatic delivery of ApoE is reduced in AQP4 null mice and after sleep deprivation [133], suggesting that the link between the glymphatic system and AD could go beyond impaired extracellular waste clearance.

The physical exercise-induced improvements in glymphatic function have been concentrated on the recovery of the aging-associated reduction of polarized AQP4 [32]. Nevertheless, the neuroprotective effects of physical exercise in dementia have also been associated with sleep regulation [134] and improvement of vascular function [98, 135]. Aerobic exercise has been especially effective in reducing sleep medication intake and sleep latency in the elderly and patients with AD [134]. Since the prevalence of sleep disorders and physical inactivity among AD subjects is remarkably high [19], the beneficial effects of physical exercise on improving sleep could also contribute to the glymphatic function. Moreover, animal studies have shown that long-term physical exercise protects against neurovascular deterioration by preventing aging-associated astrocyte dysfunction, pericyte injury, and basal lamina impairments [98, 135]. Of note, Morland and colleagues [136] demonstrated that the physical exercise angiogenic properties are dependent on the activation of the lactate receptor, hydroxycarboxylic acid receptor 1 (HCAR1), which is highly expressed in pericyte-like cells surrounding penetrating arterioles. Since the polarized expression of AQP4 relies on the pericyte-astrocyte interaction, it is possible that the increase in polarized AQP4 associated with physical exercise is preceded by the effects of exercise on pericytes. These are interesting topics that warrant further exploration.

Evidence also suggests that the glymphatic system might be further impaired as AD progresses [24, 137]. Intracerebroventricular Aβ injection induces astrogliosis and reduces the expression of polarized AQP4, resulting in impaired glymphatic activity [24]. Moreover, a recent study using a transgenic animal model of AD with tau protein overexpression reported that aged but not young transgenic mice show a reduction in glymphatic activity, suggesting a link between tau protein accumulation and damage to such clearance system [137]. Therefore, impairments in the glymphatic and meningeal lymphatic activity may precede the disease onset, while the consolidation of the AD pathophysiology can further impair the activity of the clearance systems.

Finally, enhancing the central clearance systems by increasing the glymphatic or meningeal lymphatic function may be used as an augmentative treatment rather than an exclusive intervention. In rodents, overexpression of VEGF-C followed by mAducanumab treatment (the mouse analog of Aducanumab) significantly increases Aβ clearance by immune therapy [75]. Such anti-Aβ treatment is clinically effective in counteracting AD-associated proteinopathy [106], although with limited effects on cognitive function. The potential synergistic effects of increasing the brain waste clearance function concomitant with anti-Aβ immune therapies warrants further exploration.

## Data Availability

Not applicable.

## Abbreviations

AD: Alzheimer’s disease
Aβ: Amyloid-beta
CNS: Central nervous system
BBB: Blood–brain-barrier
CSF: Cerebrospinal fluid
ISF: Interstitial fluid
AQP4: Aquaporin 4
dcLN: Deep cervical lymph nodes
PDGF-B: Platelet-derived growth factor B
PET: Positron emission tomography
NMDA: N-methyl-D-aspartate
CCR7: C–C chemokine receptor type 7
PUFAs: N-3 polyunsaturated fatty acids
NBP: L-3-n-butylphthalide
tPBM: Transcranial photobiomodulation
TMS: Transcranial magnetic stimulation
VEGF: Vascular endothelial growth factor
DSCR1: Down syndrome critical region 1
ApoE: Apolipoprotein E
HCAR1: Hydroxycarboxylic acid receptor 1
